# General practitioners’ experiences of providing somatic care for patients with severe mental illness: a qualitative study

**DOI:** 10.1186/s12875-024-02338-z

**Published:** 2024-03-22

**Authors:** Alexandra Brandt Ryborg Jønsson, John Brandt Brodersen, Susanne Reventlow, Christina Svanholm, Anne Møller, Marius Brostrøm Kousgaard

**Affiliations:** 1 Center for General Practice, University of Copenhagen, Copenhagen, Denmark; 2 Department of People and Technology, Roskilde University, Roskilde, Denmark; 3 Department of Community Health, The Arctic University of Norway, Tromsø, Norway; 4 Research Unit for General Practice, Region Zealand, Denmark

**Keywords:** General practice, SMI, Somatic care, Consultations

## Abstract

**Background:**

Patients dealing with severe mental illnesses (SMI) often face suboptimal clinical outcomes and higher mortality rates due to a range of factors, including undetected physical health conditions. The provision of care for individuals with SMI is frequently disjointed, as they engage with diverse healthcare providers. Despite this fragmentation, primary care, particularly general practitioners (GPs), assumes a pivotal role in the care of SMI patients. Our study aimed to delve into the first-hand experiences of GPs in delivering somatic care to SMI patients, concentrating on the challenges they encounter and the strategies they employ to navigate these difficulties.

**Methods:**

We conducted in-depth interviews with fifteen GPs, utilizing a semi-structured interview guide, supplemented by ethnographic observations during clinical consultations in general practice. Through inductive coding, interview transcripts and observational field notes were systematically analysed using interpretative phenomenological analysis (IPA). The findings were then deliberated upon within the author group.

**Results:**

GPs revealed that managing the chronic somatic care of SMI patients posed significant challenges. These challenges encompassed the multifaceted needs of patients, their behavior tied to symptoms, a lack of care continuity, and overarching time constraints. To tackle these challenges, the GPs had devised various strategies. However, all participants underscored the critical importance of having adequate time to properly prepare for, conduct, and follow up on consultations.

**Conclusion:**

The GPs’ interactions with SMI patients brought numerous challenges, although treating these patients were concurrently acknowledged as vital and fulfilling. The findings suggest that increased allocated time in general practice consultations for patients with SMI is important to support the somatic treatment requirements of this patient group.

**Supplementary Information:**

The online version contains supplementary material available at 10.1186/s12875-024-02338-z.

## Background

Patients afflicted by severe mental illnesses (SMI) face elevated mortality rates, translating into a shortened life expectancy by 10 to 20 years [1–3]. The heightened relative risk of suicide or accidents contributes partly to this escalated mortality, yet the majority of premature fatalities stem from somatic diseases [4]. This could be attributed to underdiagnosed and undertreated somatic diseases and cardiovascular risk factors [5]. It has been suggested that healthcare providers’ stigma against patients with SMI is a barrier to adequate access and care, and interventions are needed to reduce stigma, focusing on what matters to the individual patient [6, 7]. Also, the care provided to individuals with SMI is often marked by fragmentation, primarily stemming from the structural organization of health services and the varying regulations governing different sectors. This complexity poses challenges for healthcare professionals in effectively addressing and overseeing the entirety of the patient’s health concerns [8–11].

In Denmark, approximately 2% of the population comprises patients with SMI. All Danish citizens are affiliated with a default general practice, ensuring free and equal access to primary healthcare. Referrals through general practice extend to other primary care specialists, municipality services and hospital care [12]. Somatic diseases are primarily managed in general practice within the primary care sector. However, if patients require specialized treatment for somatic ailments, they are referred to somatic hospitals for in- or outpatient care. Patients with SMI frequently receive care across sector boundaries. Their mental health treatment may occur in hospital psychiatry, encompassing inpatient care, outpatient clinics, or specialist care in primary care. Social support, such as supported housing, is typically provided by municipal services. Despite this distributed care, general practice remains pivotal in coordinating and providing care, also for patients with SMI. General practitioners (GPs) play a central role in comprehending complex clinical scenarios, coordinating continuous care, and delivering patient-centered support for individuals and their families [13].

In various societies and healthcare systems, patients with SMI consistently face stigma, prompting a reconsideration of healthcare professionals’ approach to their care [14]. Stigma is a multifaceted issue, which is shaped by factors such as societal attitudes, limited mental health understanding, and cultural assumptions. Even in universal and free healthcare systems, like in Denmark, the challenge with stigma underscores the need for a thoughtful reevaluation of how individuals with SMI are attended to within healthcare settings. Recent studies indicate that adopting a patient-centered approach and maintaining continuity in general practice could potentially reduce the stigma and excess mortality among patients with SMI [6, 15, 16]. Nevertheless, the challenges related to potential undertreatment, underdiagnosis of somatic diseases and cardiovascular risk factors, and the resulting elevated mortality in patients with SMI remain unresolved.

Several innovative programs within primary care and general practice have aimed to address this disparity across various levels, encompassing interventions targeting risk factors and diseases on individual, health system, and socio-environmental levels [17]. However, these endeavors have yet to yield successful outcomes [17, 18]. Existing literature highlights that addressing psychosocial issues during consultations is time-intensive, and GPs often feel burdened when dealing with patients with multifaceted needs [19, 20]. Nonetheless, limited insight exists regarding the challenges GPs encounter when managing chronic somatic care for patients with SMI and the requisites to enhance care for this specific patient cohort. This study is part of a broader effort (SOFIA^1^) striving to mitigate excess mortality and elevate the need-based quality of life of patients with SMI [21]. In this article, we explore the challenges GPs confront when administering somatic care to SMI patients and the strategies they employ to navigate these complexities.

## Materials and methods

### Study design

To delve into the general practitioners’ (GPs) encounters when delivering somatic care to patients with severe mental illnesses (SMI) in general practice, a qualitative study using ethnographic approaches was undertaken. This study involved engaging in interviews with GPs and observing consultations between GPs and patients with SMI. The research encompassed a total of 15 in-depth interviews and 35 h of observation during consultations across five distinct general practice clinics situated in the Zealand and Central Denmark regions. The data collection spanned from February 2018 to March 2019, with supplementary follow-up interviews and observations conducted in September and October 2020.

### Informants

Fifteen GPs were recruited through an open call posted via the list-serve of the Research Unit for General Practice, at the University of Copenhagen and via snowball-recruiting [22]. Of these 15 GPs that agreed to participate in interview, three were randomly selected and asked by AJ to allow for observations of three consultations with patients with SMI. Additionally, three GPs who had already been giving feedback on the initial SOFIA study protocol [21] were invited to participate in the observational study by AJ.

The informants (Table 1) were selected strategically to have maximum variation in relation to gender, age, and type of clinic: solo practice (S); one GP and practice staff, company practice (C) more than one GP sharing facilities and staff.

**Table 1:** Informants

Participants	Gender	Age	Interview type	Practice type	Special interest
GP1	Female	60	I	S	FPP
GP2	Female	56	I + O	S	n/a
GP3	Male	50	I + O	C	PIP
GP4	Female	38	I	C	FPP
GP5	Female	48	I	C	NIP
GP6	Female	52	I + O	C	PIP/PCP
GP7	Male	46	I + O	C	NIP
GP8	Female	49	I	C	NIP
GP9	Male	39	I	C	PIP
GP10	Female	69	I	S	PIP
GP11	Male	55	I	C	n/a
GP12	Female	64	I	C	n/a
GP13	Male	61	I	S	PIP/PCP
GP14	Male	57	I + O	C	n/a
GP15	Male	42	I + O	C	PIP

The following “Strategic coding” was used in case of selection beyond gender / age / type of practice: Particular interest in psychiatric patients “PIP”, No (particular) interest in psychiatric patients “NIP”, Former or current practice coordinator psychiatry “PCP”, Many psychiatric patients associated with practice “MPP”, few psychiatric patients associated with “FPP”

In addition, some informants were selected based on their special interest in patients with SMI. Others were selected owing to their previous function as practice coordinators in relation to psychiatry. We also strived for variation in the sample so that both GPs with little experience with patients with SMI and GPs with much experience were represented.

### Data collection

Fifteen semi-structured interviews were conducted employing a semi-structured interview guide. Interviews were audio recorded and transcribed verbatim. The duration of interviews spanned from 40 to 75 min, and all occurred within the GPs’ clinics. The interview guide underwent minor adaptations throughout the interview process as emerging data prompted new lines of inquiry. The questions posed during the interviews were framed in an open and neutral manner. They explored aspects such as the GPs’ customary care for patients with SMI, their collaborations with other healthcare professionals, their approach to assessing patients’ somatic health, their perceptions of the consultation process, and the challenges they encountered in caring for these patients.

Additionally, the interviews were complemented by observations of clinical consultations. During these observations, notes were taken and subsequently expanded upon and analyzed in alignment with the interview transcripts. Adhering to the ethnographic tradition, the observations are expounded upon and presented as findings to offer a more profound understanding of the research subject [23]. The interviews and observations were conducted by a trained GP (CS) and an experienced medical anthropologist (AJ).

The Organization of General Practitioners in Denmark’s agreement on financial compensation for any eventual loss of income while participating in research was observed.

### Analysis

The analysis of the collected data was undertaken collaboratively by CS, AJ, and JBB, employing an interpretative phenomenological analysis (IPA) approach [24]. IPA is a qualitative research method used to explore the meanings individuals give to their experiences rather than seeking objective truths. It originated in psychology but has since been applied across various disciplines. In the analytic process, we identified emergent themes and patterns in the participants’ accounts. These themes provided insights into shared meanings and variations in how GPs interpret and make sense of their clinical experience in providing care to patients with SMI. This method thus facilitated a comprehensive exploration of the core inquiries within the study. IPA proves particularly advantageous in studies with small sample sizes, as it allows for an intricate examination of participants’ experiences at a micro-level [24]. This approach ensures that each participant’s voice is equally valued, especially when dealing with limited participant numbers.

The complete textual transcripts were subjected to multiple readings. Utilizing the NVivo software for qualitative analysis, they were systematically deconstructed into meaningful units and then coded based on the primary subjects derived from the modified interview guide. Given the substantial volume of material, this process yielded a notable number of codes relative to themes. Consequently, an initial sorting into code groups was executed. Following this, the initial themes were presented and deliberated upon within the research team. The meaningful units, having been sorted into code groups, were subsequently re-examined and categorized once more. In the conclusive phase of the analysis, the codes, selected quotes, and field notes were reviewed and discussed in connection with the original full-text transcripts. This process allowed for a re-contextualization, shaping the final version of the text for this article. For the purposes of this article, citations from the original material were translated into English. All translations including the writing of the paper have been assisted by AI tools.

### Ethics

Every participant received a letter containing comprehensive information about the study prior to their involvement, and written consent to participate was obtained from all individuals. Participation included interviews for all GPs (*N* = 15), and for a selected group of GPs (*N* = 5) it also included giving consent to being observed during a 7-hour working day and to have informal chats with researchers that could be used for analytical purposes. Patients who were subjects of observations were similarly informed about the study and were given the opportunity to decide whether they wished to participate. All patients provided their oral agreement and consent. To safeguard privacy, all GP participants’ identities were anonymized, and no identifiers on patients were obtained. Data management adhered to the guidelines stipulated by the General Data Protection Regulation (GDPR). The study adhered to the overarching principles of ethical research as delineated by the most recent version of the Helsinki Declaration. The SOFIA study received approval from the Regional Ethical Committee.

## Results

Our findings reveal that although GPs perceived the delivery of somatic care to patients with SMI as a significant and potentially impactful responsibility, they also encountered several challenges that made this task demanding. In the following sections, we outline these challenges and elucidate how GPs endeavored to address them. These challenges encompass (a) the dynamics of consultations within the practice, (b) the GP-patient relationship, covering aspects like continuity of care, and (c) the coordination of care across different sectors.

### The dynamics of consultations within the practice

#### Multiple, complex problems require extra time

The GPs emphasized that certain patients with SMI often required additional time compared to other patients due to the complexity of their concerns. An illustrative instance during an observed consultation involved a woman in her late 50s, diagnosed with schizophrenia. Despite the GP’s initial attempt to focus the discussion on her scheduled blood pressure query, she spent nearly ten minutes discussing her suspicion that her neighbor was spying on her and plotting to evict her from her apartment. In a subsequent interview, the GP revealed that he chose not to interrupt her, as he recognized that attentive listening was integral to cultivating and sustaining a rapport with this specific patient. Eventually, the patient began describing why she felt anxious about her blood pressure. She employed phrases like “*I don’t fit into my body*,” “*I feel restless*,” and “*I am feeling hot in the rain*.” These symptoms were attributed to high blood pressure, which enabled the GP to proceed with an examination. However, this led to a 20-minute delay, subsequently causing the GP to feel rushed and stressed while attending to other patients throughout the day (AJ fieldnote, GP15, Fall 2019).

The GPs found that the health concerns of patients with SMI frequently exhibited a complex nature, often intertwined with social or non-medical issues. An observed consultation offers an illustrative example: Jeanne, a 27-year-old woman diagnosed with schizophrenia, PTSD, and several somatic conditions, arrives at her GP’s office for a routine asthma check. As she enters the consultation room, a somber expression fills her eyes. Almost immediately after the GP seeks permission for AJ to observe the session, Jeanne removes her shirt and addresses AJ with a loud voice, “*I hope you’re not too prudish*.” She then turns to the GP and speaks even louder, proclaiming, “*Something is wrong, my stomach is black, see for yourself!*” Following an extended exchange and a physical examination, the GP reassures Jeanne that there’s nothing amiss with her stomach, explaining that it’s simply her veins that are visible. This assurance prompts her to burst into tears. She confides that she has lost her job and is grappling with financial uncertainty. The GP offers a brief comforting response before attempting to address her asthma check. However, she declines, stating that it’s futile due to her escalating smoking habit, admitting she’s now consuming a pack a day. Instead, she insists on receiving a prescription for sleeping pills, sharing, “*I worry so much that I can’t sleep, and you know how I get if I don’t sleep—like a crazy animal*.” The consultation, initially scheduled for 15 min, stretches to 30 min. The GP tries to schedule another session, with Jeanne agreeing as long as the discussion doesn’t revolve around her smoking. Subsequently, the GP tells AJ that he has substantial concerns about Jeanne’s somatic health but currently prioritizes establishing regular visits to monitor her well-being. The observation ends (AJ observation GP14, Fall 2019).

Due to challenges with continuity, as we will elaborate on subsequently, the GPs often lacked a comprehensive understanding of all their patients with SMI. As a result, additional time was occasionally necessary before or during a consultation to familiarize themselves with the patient’s medical history. While a significant number of patients with SMI indeed necessitated extended consultation times, one GP noted an alternative scenario. Some patients with SMI struggled to endure consultations lasting over 10 min due to heightened anxiety or paranoia. This circumstance imposed pressure to swiftly discern the essential aspects within a limited timeframe.

#### The risk of overshadowing

The time constraints during consultations could exacerbate challenges in accurately discerning whether the patient’s symptoms were attributable to a somatic condition, medication side effects, or linked to their mental illness. This could increase the risk of diagnostic overshadowing, a ‘negative bias impacting a clinician’s judgment regarding co-occurring disorders in individuals who have intellectual disabilities or other mental illness’ [25]. Preventing overshadowing was said by GPs to be a more time-intensive process (both within and after the consultation) yet deemed essential for accurate somatic disease diagnosis.

*“It’s referred to as overshadowing, where mental symptoms mimic somatic symptoms. It’s crucial not to dismiss these symptoms by attributing them solely to the mental illness but take them seriously and do a physical examination”* (GP 3).

Some GPs shared instances where overshadowing was prevalent among other medical specialists, necessitating a significant investment of time from the GPs in communicating with the doctors and nurses whom they had referred patients to. The objective was to clarify that the reasons for referral were indeed rooted in somatic issues and not the mental illness.

#### Challenges with adherence

Another factor contributing to the complexity and challenge of consultations was patients exhibiting poor adherence to treatment plans. Challenging life circumstances often hindered some patients from adhering to prescribed treatments, and this was perceived as a risk by the GPs:

“*Among those with schizophrenia, many younger patients will be on multiple medications, coupled with elevated cholesterol levels, obesity, and smoking. This presents a considerable risk. While they might not presently have any somatic disease, there are instances where altering their behaviour could save lives*.” (GP8).

The GPs emphasized that patients within this group commonly lacked recognition of their own illnesses. Frequently encountered symptoms like paranoia could impede adherence to treatment regimens, such as taking medications. For instance, if patients suspected the GP to be involved in a conspiracy against them, it was difficult for them to comply. In such scenarios, the GPs noted that patients required assistance with dosage and reminders. Additionally, one GP elucidated how the combination of health and social challenges often influenced a patient’s capacity to prioritize health issues:

“*Patients always have something that they see as most important in their lives. People often wonder why they can’t just eat healthier or take better care of themselves. Healthy food isn’t always expensive – there are many nutritious options that don’t cost much. However, it’s not easy to address issues like obesity if you don’t have support and love in your life. When you’re constantly worried about things like paying rent or dealing with problems at home, focusing on your health can become very difficult. It’s similar with mental illness. If you’re struggling emotionally, it’s really hard to make big changes in your lifestyle.*” (GP2).

GPs also encountered varying situations concerning patients’ adherence, highlighting the diverse nature of the patient group:

“*Not everyone’s mental illness causes cognitive impairment. I have a patient who’s dealing with a lot – he abuses drugs, had strokes, has memory problems, anxiety, and more. It’s really challenging to help him because he struggles to follow the treatment plan. On the other hand, I have a patient with paranoid schizophrenia. He’s married and has a family that supports him, even though it’s tough for them sometimes. He’s doing a good job of taking care of his diabetes, coming to appointments, and seeing his psychiatrist. So, I would say he’s making good progress overall.*” (GP11).

#### Insufficient time allocation

As noted by the participating GPs, a prevalent challenge was the lack of time available to meet the needs of all their patients, particularly those dealing with more intricate health concerns. The abovementioned factors underscored the reality that consultations involving patients with SMI often demanded more time than the standard 10-15-minute appointments. Despite this, most of the GPs involved did not allocate extra time for consultations with SMI patients. This was partly due to the need to attend to their other patients as well, and partly because reimbursement was only provided for regular consultations, as stipulated by the collective agreement at the time of the study:

“*I wish there was an option to be compensated for 30-minute consultations with the most complex patients*.” (GP3).

Furthermore, the insufficiency of (reimbursed) time to properly follow up with patients who had received care from providers in other sectors was perceived as a hindrance to delivering comprehensive care.

### The GP-patient relationship

All the GPs emphasized the crucial role of establishing a trusting relationship between themselves and the patient for delivering effective care. However, cultivating such relationships with patients who have SMI could often be challenging due to issues related to continuity, accessibility, and the patients’ behaviors in social interactions.

#### Challenges with continuity and reach

Certain patients with SMI enjoyed a long-standing and dependable doctor-patient relationship within the same practice, built over several decades. However, other patients with SMI exhibited a pattern of frequently changing their general practices. According to the GPs, such shifts could arise from conflicts with the staff, or in cases where individuals with substance use challenges were denied new prescriptions. There were also instances where patients relocated to different towns to evade social repercussions from local authorities. Additionally, some patients only had access to substitute GPs due to residing in areas where a scarcity of general practitioners prevented assignment to a regular practice. These circumstances collectively contributed to a lack of continuity in the GP-patient relationship. This fragmentation in the relationship raised concerns among GPs about potential underdiagnosis or undertreatment of somatic diseases. The lack of consistent follow-up on symptoms and treatment due to this discontinuity posed significant worries for the GPs.

“*There has to be someone who takes charge, someone who’s responsible for overseeing diabetes treatment. We also need to monitor for signs of cancer (…) Otherwise, there won’t be anyone noticing if something isn’t right (…) This is particularly important because patients with severe mental illnesses often struggle without consistent.*” (GP8).

Several GPs had encountered situations where patients didn’t receive any treatment at all due to being “lost in the system”:

“*It’s like we lose track of the patient. I don’t feel guilty, but they seem to slip through the cracks and we can’t… we need a better way to stay connected and follow up on this, the current approach isn’t effective.*” (GP4).

Moreover, when patients with SMI were new to the practice, GPs often encountered difficulties in acquiring comprehensive information about them:

“[To get patient information] *You’d have to reach out to 4–5 different general practices because these patients are often changing locations, and no one really knows them or remembers their details. This makes it quite challenging.”* (GP9).

A majority of GPs indicated that they likely had more patients with SMI linked to their practice than they were aware of. This was due to the fact that some patients with SMI might never seek medical attention from their GP. Even when these patients did reach out to the practice, they frequently failed to attend their scheduled appointments:

“*Their attendance is unpredictable, like the wind shifting directions (…) Trying to schedule an appointment feels almost impossible (…) It’s not that they don’t want to come, it’s just that they struggle to overcome the challenge of making it to an appointment on that specific day.*” (GP13).

When patients didn’t attend their scheduled appointments, GPs found it challenging to offer sufficient somatic care. In certain cases, alternative methods were employed to connect with these patients:

*“We can tell when she’s not feeling well because she misses appointments. So, we make an effort to reach out to her. If that doesn’t work, we might need to contact her sister, who she lives near and sometimes stays with.”* (GP2).

#### Volatile social relationships

According to the GPs, individuals with SMI frequently experience unstable social relationships. This phenomenon extends to their interactions with their GP and other healthcare providers. The fluctuating nature of these relationships occasionally posed challenges for GPs in building a foundation of trust, thereby impacting their ability to offer comprehensive care for somatic diseases. The volatility of these relationships was viewed as an intrinsic aspect of living with SMI. Recognizing and acknowledging this dynamic was considered vital by the GPs.

*“Recognizing their symptoms is crucial, and it’s important to assist them in navigating the healthcare system effectively.*” (GP3).

All GPs unanimously concurred that both the GP and the entire general practice clinic should make a dedicated effort to provide assistance to patients with SMI:

“*We’re his only support, but if we pressure him too much, he might stop coming altogether.*” (GP13).

However, this could pose challenges, particularly when the behavior of the patients was problematic:

“*To effectively handle things, you need to have a good handle on your own emotions and maintain a sense of balance.*” (GP2).

Occasionally, a patient with SMI would exhibit threatening behavior or be at risk of becoming violent. In such situations, GPs needed the capability to trigger an alarm or have an escape route ready. However, they emphasized that conflicts, although infrequent, often occurred in the waiting room. The GPs expressed more concern for the safety of their staff. As a countermeasure, these patients would receive what one GP referred to as “VIP treatment.” This approach involved providing patients with special needs certain privileges and advantages in comparison to regular patients:

*”When Johnny comes to the front desk, the secretary offers him a cup of coffee, and then one of us sees him as soon as we have an opening. If we don’t do this, he might not return for the next six months.”* (GP6).

A GP noted that some patients exhibited behavior known as “splitting,” leading the practice to formulate a shared policy for managing such situations:

*“In our clinic, we share a common goal: providing specialized care to ensure equitable treatment for them* [patients with SMI]. *Additionally, we have specific protocols in place to address situations where patients might attempt to create conflicts among the staff. We enforce strict regulations to prevent such issues from arising.”* (GP2).

Another potential source of conflict could arise from issues related to addiction among the patients. One GP had established specific guidelines for patients with SMI, which they were required to adhere to in order to receive treatment at the clinic:

“*There’s one condition I make clear right away: they cannot be addictive to illegal substances. That’s the first thing I communicate. Without adhering to this, we won’t be able to provide treatment. It’s the choice they have to make. I can’t assist them if they continue to misuse substances.*” (GP9).

This quotation did not align with findings from other interviews. Instead, the emphasis was on the recognition that numerous patients with SMI were contending with a challenging use of illegal substances. It was underscored that this aspect should be considered as an integral part of the overall picture. In line with this, a holistic approach was noted to be especially pertinent for patients with SMI due to the intricate interplay of their mental, somatic, and social challenges. This intricate dynamic was observed to impact their treatment, adherence, and overall outcomes:

“*This patient has endured significant mistreatment – it’s hard to fathom. This has happened because no one has truly listened to her or considered her experiences. If her story had been taken into account, she might not have reached such a severe state of illness. There’s a lack of understanding about her situation.*” (GP2).

Consequently, having an understanding of each patient’s individual difficulties was crucial in determining an effective treatment strategy. This encompassed cultivating a trustworthy rapport with the patients’ family members, if possible:

“*Getting to know the patient has a remarkable impact, and it’s worth mentioning that building a relationship with their family members also contributes to this positive outcome.*” (GP4).

When examining the dynamics between GPs and their patients with SMI, we observed that challenges stemming from the GPs themselves (such as their attitudes towards the patients) were not explicitly raised in individual interviews. However, casual conversations with GPs after the interviews often unveiled sentiments that indicated some found patients with SMI occasionally ‘exhausting,’ ‘burdensome,’ ‘annoying,’ ‘disgusting,’ ‘demanding,’ or ‘difficult to connect with.’ These sentiments were not part of the data collection; however, CS retrospectively recounted them during the analytical discussion. We revisit this when discussing the study’s limitations.

### The coordination of care across different sectors

#### Communication shortcomings and insufficient somatic care in psychiatry

A lack of effective communication regarding treatment between hospital psychiatry, psychiatric residences, and at times, subpar somatic care within these settings, presented hurdles to GPs in providing optimal patient care. When patients were admitted to psychiatric wards for extended periods, the responsibility for somatic care fell on the ward. However, GPs frequently encountered instances where this care was deemed inadequate:

“*At times, the psychiatric ward handles somatic care, but there are instances when years go by without it being addressed. I’ll inquire with the patient, asking, ‘Have you had your blood samples taken?’ and it often turns out that they haven’t been checked for years.”* (GP7).

GPs elucidated that they frequently became aware of these lapses in somatic care when patients contacted them for prescription renewals. A GP shared that, at times, there appeared to be no cohesive treatment coordination due to a lack of communication among psychiatric wards. Consequently, she would only gain insights into her patients’ treatments by reviewing their medication records:

“*There are moments when I’m unaware if the patients are still receiving treatment at the psychiatric wards, only finding out when they contact me to renew their prescriptions.*” (GP10).

Certain GPs noted that they didn’t receive updates from outpatient psychiatry until the patient’s discharge, which could span several years. Additionally, collaboration hiccups emerged with social psychiatric residences regarding residents’ somatic care, attributed to inadequate competencies among the residence staff. A few GPs illustrated this with instances where social workers lacked the ability to measure blood pressure or reached out to the GP clinic with inquiries outside the scope of the GPs’ responsibilities. This subsequently escalated workload within general practice.

#### Shifting referral regulations

Numerous GPs highlighted the continual modifications in referral regulations as a significant hurdle in managing the well-being of patients with SMI. To illustrate, during one of the observed consultations between a GP and a patient diagnosed with schizophrenia and cardiovascular disease, accompanied by his sister, the patient sought a referral for psychiatric care. Despite experiencing a deterioration in symptoms, as confirmed by his sister, his previous referral had been rejected because the local regional psychiatrist no longer accepted patients with schizophrenia. The patient now required a referral for hospital psychiatry. This visibly frustrated the GP, who later referenced this incident during an interview, stating:

*“It’s like navigating a chaotic and unpredictable environment. The regulations for referrals keep changing, and I have no way of staying updated. This ends up being a waste of both the patient’s and my time.*” (GP14).

Furthermore, a notable number of GPs emphasized that gaining acceptance for referrals in the secondary care sector had become progressively challenging. As a consequence, they frequently experienced a sense of inadequacy when dealing with patients whose referrals were turned down.

## Discussion

Patients with SMI face elevated mortality rates partly due to undetected somatic ailments and cardiovascular risk factors, compounded by treatment and adherence difficulties. As a result, identifying health issues, risk factors, and enhancing treatment efficacy, are pivotal. Nonetheless, the findings of this study shed light on numerous hurdles in delivering somatic care to patients with SMI within general practice. Hence, the intricate nature of these patients’ challenges renders consultations in general practice more intricate and time-intensive, encompassing the diagnosis of new health issues and the continued management of existing ones. Several facets contribute to this complexity. First, patients often grapple with a blend of somatic, mental, and social predicaments, necessitating additional time for symptom presentation. Second, the patients’ challenging circumstances can disrupt continuity of care, straining the doctor-patient relationship. Alas, it might also occur that the general practitioner declines to treat patients actively using illegal substances. Likewise, patient-initiated discontinuity of care may be a result of conflicts related to prescription drugs or challenging use of illegal substances. Additionally, GPs observed that coordinating and communicating across different healthcare sectors posed care-related obstacles, also demanding supplementary time. These diverse challenges may prompt GPs to perceive caring for patients with SMI as demanding, aligning with research revealing a link between underserved patients and GP burnout [12].

### Diagnosing (new) diseases and risk factors in the consultation

In general practice, consultations encompass a spectrum of scenarios involving both previously diagnosed conditions and new, potentially undetected ailments. Both patients with and without SMI may encounter challenges that lack straightforward solutions. For most patients without SMI, well-documented medical records provide GPs with insights into their medical histories, and there exists an established patient-GP rapport. However, our findings reveal that this isn’t always the case for patients with SMI, owing to disruptions in continuity of care.

When it comes to the intersection of SMI and somatic comorbidity, diagnosing new conditions becomes a distinct challenge. GPs in this study noted that patients often required additional time to convey a range of symptoms, and that they, as GPs, needed more time to synthesize these symptoms within their diagnostic process. As highlighted by Christensen et al. [26], patients with SMI often struggle to differentiate between symptoms and evaluate which ones could signal new illnesses. This observation resonated in our study, where patients frequently encountered difficulties articulating and explaining their physical symptoms. Collectively, these factors elevate the risk of underdiagnosing (and subsequently undertreating) patients with SMI, a concern also flagged in existing literature [27].

Molloy et al. [27] describe the multitude of factors contributing to overshadowing and consequent underdiagnosis. These include aspects of the clinical environment (like privacy concerns and time constraints), health professionals’ limited mental health expertise, stigma-based attitudes, and patients’ challenges in effectively communicating their physical healthcare needs. In our study, certain GPs were cognizant of the risk of overshadowing and how symptoms from various illnesses might intertwine. Adding to this complexity, individuals with SMI frequently encounter prejudice and societal marginalization [28], fostering distrust in authorities, including healthcare personnel. This further impedes the nurturing of a robust patient-GP relationship [29, 30]. To counteract the underdiagnosis and undertreatment of somatic diseases and associated risk factors among patients with SMI, addressing concerns such as symptom presentation, overshadowing, and distrust is imperative within the consultation process.

### Time allocation

The findings from this study suggest that extended time allocation in consultations with SMI patients could support GPs in managing the unique needs of patients with SMI. Previous research has similarly highlighted that consultations dealing with both psychological and somatic health issues are inherently more intricate and demand more time than standard consultations [20]. This prompts a reevaluation of reimbursement models to accommodate lengthier consultations in general practice tailored to patients with SMI. In alignment with this notion, and inspired by insights from the SOFIA study, the Danish Regions and The Danish Organisation of General Practitioners have recently instituted an agreement (effective from January 1st, 2022) that includes reimbursed annual extended consultations for patients with SMI. It is worth noting, however, that the perception and utilization of this additional time in GP consultations can differ between GPs and patients [31]. Therefore, it is important to explore how GPs and patients both view the value of this extended time. This encompasses aspects such as nurturing a stronger doctor-patient relationship, dedicating time to address non-medical concerns, enhancing need-based quality of life, acknowledging subjective inequities, discussing physical symptoms, and conducting thorough physical examinations [21].

While the integration of more time into general practice consultations offers a promising avenue for enhancing somatic care for patients with SMI, our results, alongside previous research, propose that considerable challenges will persist. These challenges arise from the patients’ mental health conditions, their often challenging life circumstances, and fragmented care delivery. For instance, a study on patients’ perspectives on illness and self-care illuminated how, for individuals with SMI, their mental symptoms can overshadow attention to their somatic conditions [32]. Organizational factors were examined in an intervention involving integrated care for patients dealing with stress, anxiety, and depression. However, the outcomes were unsatisfactory [33], leading to the decision not to include the integrated care model in the SOFIA study [21]. Furthermore, investigations into the implementation of health promotion in social psychiatric residences reveal that professionals confronted multifaceted barriers related to patients’ intricate situations, behaviors, and diverse needs and capacities. As a result, staff members had to tailor activities to suit individual circumstances, necessitating a high degree of interpersonal engagement, flexibility, and additional time [34]. Consistent with our study’s findings, recent research also underscores the presence of various organizational hurdles in delivering holistic, cross-sectoral care to patients with SMI [8–10]. Contextual factors encompassing patients’ living conditions and the structures of health and social care systems are beyond the direct control of individual GPs. Thus, future interventions aimed at enhancing somatic care for patients with SMI should encompass a broader perspective, considering the intricate interplay of societal and organizational dynamics [9, 35].

### Limitations of the study

During the course of our study, an interesting observation emerged regarding the challenges raised by the interviewed GPs in relation to treating patients with SMI. What struck us was that the GPs predominantly highlighted challenges that seemed to be external to their own role and perspective as medical practitioners. This was particularly intriguing because we were aware of previous instances in other research projects where GPs had openly expressed feelings of patients with SMI being demanding or burdensome. Additionally, research from other countries had indicated that general medical clinicians might harbor negative attitudes toward individuals with SMI [36].

Upon closer examination of the interview transcripts, it appeared that the GPs, who were conversing with a fellow colleague, might have tailored their responses to portray a higher level of engagement and empathy toward this specific patient group. In order to minimize any potential influence from the GP-interviewer dynamic and create an environment for more candid conversations, we diversified our approach by having a trained anthropologist conduct the remaining interviews and observations. When queried directly, the GPs mentioned feeling a sense of confidentiality and ease when discussing complex matters with a peer. However, the responses obtained from interviews conducted by the anthropologist tended to exhibit more candid and negative perspectives. While it’s uncertain whether this observation pertains to a broader methodological issue or is specific to the interplay between our interviewers and interviewees, it does raise the possibility that not all the challenges pertaining to somatic care for patients with SMI were fully brought to light in our study.

Additionally, it’s worth noting that the GPs’ diverse experiences and backgrounds likely played a role in shaping their perceptions of what constitutes challenges in this context. To maintain transparency, we have incorporated information about the GPs’ prior experiences and interests in Table 1. It’s also essential to acknowledge that this is a relatively small-scale interview study, involving 15 GPs. While we aimed for data saturation, a larger participant pool might have enabled further analysis to uncover differences in backgrounds, experiences, and even gender among the GPs.

## Conclusion

This study delved into the first-hand encounters of general practitioners (GPs) as they administered somatic care to patients with severe mental illnesses (SMI). The GPs’ interactions with SMI patients brought forth numerous challenges, though treating these patients were concurrently acknowledged as vital and fulfilling. The insights gleaned from this exploration warrant careful consideration during policy formulation to ensure that both financial and organizational prerequisites more comprehensively facilitate GPs in delivering somatic care to SMI patients and counteract potential burnout among GPs. Within the realm of Danish general practice, recent strides have been taken towards this end, evident in the implementation of extended consultations tailored for SMI patients. Nonetheless, contextual obstacles rooted in the life circumstances of patients and the intricate organization of cross-sectoral care continue to persist. Finally, patients with SMI encounter stigmatization and face poor health outcomes, regardless of the healthcare system or the extent of care fragmentation. This underscores the broader relevance of the study.

### Electronic supplementary material

Below is the link to the electronic supplementary material.

Supplementary Material 1

## Data Availability

Participants have not consented to data being shared. Further, data is not applicable to sharing because it will risk breaking the anonymity.

## Footnotes

1 “Sammen Om Fysisk og psykisk helbred I Almen praksis” (SOFIA), in English: “Joint Forces on Somatic and Mental Health in General Practice”.
