# Supplementary material for: General practitioners’ experiences of providing somatic care for patients with severe mental illness: a qualitative study
Source: BMC Prim Care. 2024 Mar 22;25:96. doi: 10.1186/s12875-024-02338-z (PMC10958876; doi:10.1186/s12875-024-02338-z)
Supplement: Supplementary file 1 — Supplementary Material 1 [file 12875_2024_2338_MOESM1_ESM.pdf]

Interview Guide for Semi-Structured Individual Interviews

**Presentation (2 min)**		
	Presentation of the interviewer	Name, education, practice
	Timeframe and compensation	The interview will last approximately 1 hour, and you will receive 926.8 DKK for your participation – Compensation will be provided by sending an invoice via virk.dk (provide guidance)
	Data storage	All information will be treated strictly confidentially and stored securely, in accordance with the Personal Data Protection Act.
	Consent	Can you confirm that you have been informed about the study and that you wish to participate?
**Presentation of the Project and its Purpose (5 min)**	Presentation of the Phy-Psy Trial	People with severe mental illness (SMI) have a life expectancy 10-20 years shorter than the general population. Recent research has shown that excess mortality can be reduced by establishing a structured and individualized treatment in general practice.<br><br>"The Phy-Psy-Trial" is a major research project conducted by the Research Unit for General Practice in Copenhagen, sponsored by the Novo Nordisk Foundation.<br><br>The primary purpose is to develop a treatment model that can identify chronic physical illnesses in patients with SMI and potentially reduce their excess mortality.
	Purpose of the Interview	I need your help to gain insights into and understanding of the everyday practices in general practice concerning the treatment of severely mentally ill patients with concurrent physical illnesses. This knowledge will be used in the future to develop a better treatment model to reduce excess mortality in this patient group.
**Additional Information about the Informant (5 min)**		Inquire about any previous interest in mental illness, comorbidities, or other topics that need further elaboration based on the responses in the survey.
**Interview Questions**		

Description of Current Practices	
Question 1	Do you have many patients with mental illness?
Question 2	Do many of them also have physical illnesses?
Question 3	How do you manage their psychiatric medications? (with assistance from others?)
Question 4	What role do you believe their mental illness plays in the consultation?
Question 5	What are your thoughts on their compliance? (including attendance)
Question 6	Do you involve their family members?
Question 7	Do you use clinical guidelines for mental/physical illnesses? (why/why not?)
Question 8	What role does your staff play in handling these patients?
Question 9	What is your personal experience with consultations with patients with mental illness?
Assessment and Analysis	
Question 10	What do you see as the specific challenges in the treatment of these patients?<br>For the patients<br>For the your practice<br>For you personally
Question 11	Can you provide examples of patients or situations where your treatment of SMI patients has been successful?
Collaborators	
Question 12	How is your collaboration with the municipality? (Support workers, social psychiatry, etc.)<br>Who do you have contact with/Who represents the municipality for you?<br>Do you have an overview of the services? Are they adequate?<br>What challenges do you see?<br>Do you have suggestions for changes?
Question 13	How is your collaboration with medical specialties? (Outpatient clinics, specialists, etc.)<br>Who do you have contact with?<br>Do you have an overview of the services? Are they adequate?<br>What challenges do you see?<br>Do you have any suggestions for changes?
Question 14	How is your collaboration with psychiatric services?<br>Who do you have contact with?<br>Do you have an overview of the services? Are they adequate?<br>What challenges do you see?<br>Do you have suggestions for changes?
Question 15	

	How is your collaboration with clinical pharmacologists or pharmacists?<br>Who do you have contact with?<br>Do you have an overview of the services? Are they adequate?<br>What challenges do you see?<br>Do you have suggestions for changes?
Assessment and Analysis	
Question 16	What do you see as the specific challenges in the collaboration between SMI patients and you?<br>For the patient?<br>For the practice?
Question 17	Can you provide examples of collaboration or organizational aspects that have worked well with patients with mental illness?
Suggestions for Intervention?	
Question 18	Do you have ideas for a treatment model for patients with mental illness and concurrent chronic physical illness? (Such ideas may be wild)
Question 19	Do you have suggestions for a model for collaboration with other stakeholders (municipality, psychiatry, medical specialties, and pharmacologists) - or any other wild ideas)
Question 20	Do you see IT playing a role in these collaboration models?
(5 min)	Is there anything else you would like to add or come to think of?
**Summary (5 min)**	Summary/elaboration of field notes. The informant is asked to confirm if what has been noted down is correctly understood.
**Conclusion (2 min)**	Thank you for your time. May I contact you if there are any doubts or if further clarification is needed during transcription? Do you wish to receive future information about the project?
